# Characteristics, Management, and Outcomes of Patients Hospitalized for Heart Failure in China: The China PEACE Retrospective Heart Failure Study

**DOI:** 10.1161/JAHA.119.012884

**Published:** 2019-08-21

**Authors:** Yuan Yu, Aakriti Gupta, Chaoqun Wu, Frederick A. Masoudi, Xue Du, Jian Zhang, Harlan M. Krumholz, Jing Li, the China PEACE Collaborative Group

**Affiliations:** ^1^ The China PEACE Collaborative Group: NHC Key Laboratory of Clinical Research for Cardiovascular Medications National Clinical Research Center of Cardiovascular Diseases Fuwai Hospital National Center for Cardiovascular Diseases Chinese Academy of Medical Sciences and Peking Union Medical College Beijing China; ^2^ Central China Subcenter of the National Center for Cardiovascular Diseases Henan People's Republic of China; ^3^ Division of Cardiovascular Medicine Columbia University Medical Center New York NY; ^4^ Center for Outcomes Research and Evaluation Yale‐New Haven Hospital New Haven CT; ^5^ Division of Cardiology University of Colorado Anschutz Medical Campus Aurora CO; ^6^ State Key Laboratory of Cardiovascular Disease Fuwai Hospital Heart Failure Center Chinese Academy of Medical Sciences and Peking Union Medical College National Center for Cardiovascular Diseases Beijing People's Republic of China; ^7^ Section of Cardiovascular Medicine Department of Internal Medicine Yale University School of Medicine New Haven CT; ^8^ Department of Health Policy and Management Yale School of Public Health New Haven CT

**Keywords:** acute heart failure, characteristics, China, hospitalization, outcomes, outcomes research, quality of care, Clinical Studies, Heart Failure, Quality and Outcomes

## Abstract

**Background:**

Heart failure (HF) is an emerging epidemic in China and accounts for significant healthcare resource utilization in the inpatient setting. To create evidence‐based, life‐saving, and cost‐saving hospitalization systems, the first step is to characterize the contemporary national landscape of inpatient HF care.

**Methods and Results:**

In the China PEACE 5r‐HF study (China Patient‐centered evaluative Assessment of Cardiac Events Retrospective Study of Heart Failure), we used 2‐stage random sampling to create a nationally representative cohort of 10 004 admissions for HF from 189 hospitals in 2015 in China. Data on patient characteristics, management, and outcomes were obtained through centralized medical record abstraction. The median age of the cohort was 73 years (interquartile range, 65–80), and 48.9% were women. More than half (56.2%) of the patients were hospitalized in rural areas. Prevalence of ejection fraction ≥50%, 40% to 50%, and <40% was 60.3%, 17.7%, and 22.0%, respectively. We identified substantial gaps in care, including underutilization of diagnostic tests such as echocardiograms (63.6%), chest imaging (75.2%), and biomarker testing (56.4%), low prescription rates of guideline‐recommended medications during hospitalization and at discharge, suboptimal rates of follow‐up appointments (24.3%), and widespread utilization of traditional Chinese medicine (74.8%). The combined rate of in‐hospital mortality and treatment withdrawal in our study was 3.5%, and median length‐of‐stay was 9 days (interquartile range, 7–13).

**Conclusions:**

Patients admitted with acute HF in China have distinctive epidemiology and receive substandard care, but have low inpatient mortality despite long length of stay. These findings provide opportunities for streamlining efficiencies while improving quality of inpatient HF care in China.

**Clinical Trial Registration:**

URL: https://www.clinicaltrials.gov. Unique identifier: NCT02877914.

**Clinical Perspective**

**What Is New?**

- Substantial gaps between real‐life practice and guideline‐recommended care for patients admitted with acute heart failure exist in China, including underutilization of diagnostic tests, low prescription rates of guideline‐recommended medications during hospitalization and at discharge, suboptimal rates of follow‐up appointments, and widespread utilization of traditional Chinese medicine.
- Patients admitted with acute heart failure in China have a longer length of stay and relatively low inpatient mortality compared with Western countries.

**What Are the Clinical Implications?**

- Given the emerging epidemic of heart failure in China, we underscore the urgent need to implement tools and strategies to improve adoption of evidence‐based care for heart failure in China, as well as to streamline efficiencies.

## Introduction

Heart failure (HF) affects more than 4 million individuals in China,^1^ and ≈500 000 new cases are diagnosed every year.^2^ With the increasing prevalence of coronary artery disease,^3^, ^4^ hypertension,^5^ and an aging population,^6^ incidence and prevalence of HF in China are projected to increase further, similar to many other low‐ and middle‐income countries.^7^ In the face of this HF epidemic, there is a need to characterize the patients, their management, and their outcomes in order to develop strategies to ensure the delivery of good‐quality and cost‐efficient care.

Despite the growing importance of HF in China, there is a paucity of representative information about these patients. An important first step is to determine who is being hospitalized, what care they are receiving, and what outcomes they are experiencing. Furthermore, common to both international^8^, ^9^ and Chinese HF guidelines,^10^ there are clearly defined evidence‐based recommendations for inpatient HF care, which can be used to evaluate the patterns of care. A few previous studies suggested that guideline‐directed medications for HF were underutilized in China.^11^, ^12^, ^13^, ^14^ However, these studies were limited by their small size and mostly included only urban or tertiary‐care hospitals, thus precluding national‐level inferences.

Accordingly, we developed the China PEACE 5r‐HF (China Patient‐centered evaluative Assessment of Cardiac Events Retrospective Study of Heart Failure) study, a nationally representative sample of HF admissions in China in 2015, to characterize the demographic and clinical features, management, and outcomes of patients hospitalized for HF. This information can establish the status of hospital care of HF in China, identify gaps in care, and support future initiatives, including resource allocation and performance measurement, and ultimately translate to improved outcomes for HF in China.

## Methods

The study materials have been made available to other researchers for purposes of replicating the procedure.^15^ It is our goal to share the China PEACE prospective 5r‐HF study data; however, at this time, we are unable to do so.

### Study Design

The design of the China PEACE‐Retrospective HF study has been described in detail previously.^15^ Briefly, we used a 2‐stage random sampling design to create a nationally representative sample of admissions for HF in China in 2015. In the first stage, we identified all nonmilitary hospitals providing inpatient care for acute HF in China. We excluded prison hospitals and hospitals without a cardiovascular disease division. We also excluded traditional Chinese medicine hospitals because they typically do not provide care to patients with acute HF. We stratified the remaining hospitals into 5 economic‐geographical regions (Eastern rural, Central rural, Western rural, Eastern urban, and Central‐Western urban). We used these groups because hospital volumes and clinical capacities differ between urban and rural areas and among the official economic‐geographical regions (Eastern, Central, and Western) of mainland China. We grouped the Central and Western urban regions together because income and health services capacity per person are similar in these areas. We randomly sampled tertiary and secondary urban hospitals and all central rural hospitals.

In the second stage, we used systematic random sampling to select patients hospitalized for HF from the local hospital database of each sampled hospital. We identified cases hospitalized for HF according to *International Classification of Diseases, Clinical Modification* codes revision 10 (I50.xx, I11.0x, I13.0x, or I13.2x), when available, or through principal diagnosis terms at discharge. Central abstraction of data was performed by 2 contracted vendors using standard data definitions.^15^ We performed monitoring at each stage including case‐searching, sampling, chart‐scanning, data abstraction, and cleaning to ensure good data quality. To ensure accuracy of medical records abstraction that exceeds 98%, we used double entry for data elements that did not require interpretation (eg, admission face sheet, laboratory test results, or physician orders) and double auditing for those components requiring interpretation, such as admission note, daily progress notes, procedure notes, and discharge summary.

### Participants

We included patients aged ≥18 years hospitalized between January 1, 2015 and December 31, 2015 with a discharge diagnosis of HF. We excluded patients with a principal admission diagnosis of acute myocardial infarction (AMI). We observed that patients with chronic HF who are hospitalized in China could have HF documented as 1 of their discharge diagnoses even if acute decompensated HF was not the primary reason for their admission. As such, for further specificity, we required that patients had typical symptoms or signs of acute HF at admission and that they received typical HF treatments, including diuretics or inotropes, during hospitalization and excluded those with documented New York Heart Association class I symptoms. HF typical symptoms included orthopnea, paroxysmal nocturnal dyspnea, dyspnea at rest, dyspnea on exertion, and edema/oliguria. Typical HF signs included jugular venous distension, hepatojugular reflux, pulmonary rales, S3 gallop, and lower extremity edema.

Informed consent was waived for patients were not involved in the recruitment or conduct of the study. The Central Ethics Committee at the Chinese National Center for Cardiovascular Diseases and Yale University approved the study. All collaborating hospitals accepted the central ethics approval with the exception of 15 hospitals, which obtained local approval by their internal ethics committees. The study is registered at www.clinicaltrials.gov (NCT02877914).

### Data Collection

We abstracted data about demographics, precipitating factors, clinical characteristics at admission, diagnostic tests, and treatments during hospitalization. Information about the medical record abstraction has been described in detail previously.^15^

The New York Heart Association class for symptoms of HF at admission was identified from clinical documentation in admission notes. We collected all documented left ventricular ejection fraction (LVEF) assessed by ultrasonic echocardiogram during hospitalization and no longer than 1 month before admission. Medical history and comorbidities (including both cardiac and noncardiac) were obtained from documented history in the admission notes, discharge diagnosis, or positive laboratory test results. For example, dyslipidemia^16^ was defined as diagnosis of dyslipidemia or total cholesterol >5.18 mmol/L or low‐density lipoprotein ≥3.37 mmol/L or high‐density lipoprotein <1.04 mmol/L in men or <1.30 mmol/L in women. Anemia was defined as diagnosis of anemia or hemoglobin <120 g/L in men or <110 g/L in women.

### Medical Therapy

We utilized admission notes to identify medications taken by patients before admission. For treatments administered during the hospitalization, we abstracted information from the physicians’ orders and progress notes. Instructions and medications at discharge were abstracted from discharge notes and physicians’ orders. We evaluated the use of beta‐blockers, angiotensin‐converting enzyme inhibitors (ACEIs) or angiotensin receptor blockers (ARBs), aldosterone receptor antagonists, and anticoagulants during hospitalization and discharge among eligible patients (Table S1). We also examined the use of traditional Chinese medications in the 8 main categories used for cardiovascular disease (Data S1).

### Outcomes

We assessed in‐hospital complications, including venous thromboembolism (deep venous thrombosis or pulmonary embolism), myocardial infarction, cardiogenic shock, ischemic or hemorrhagic stroke, and bleeding from documentation in the medical charts (Data S2). We also determined in‐hospital outcomes including length of stay (LOS), death, or withdrawal from treatment because of terminal status at discharge (referred to as treatment withdrawal). Treatment withdrawal occurs in China because many patients prefer not to die in the hospital. The Chinese Government uses in‐hospital death or treatment withdrawal as a quality measure for hospitals.^17^, ^18^ Two physicians in the coordinating study center independently adjudicated all the above outcomes on the basis of patients’ clinical manifestations, results of diagnostic tests, and medical charts. In case of disagreements, they reached consensus after discussion with a cardiologist (Yuan Yu). The interobserver agreement was >98%.

### Statistical Analysis

We plotted distribution of systolic blood pressure, LVEF, and glomerular filtration rate in strata according to age groups (<35, 35–54, 55–64, 65–74, 75–84, and >85 years), sex, ethnicity (Han, non‐Han), or 5 predefined economic‐geographical regions. We reported proportions to describe categorical variables and medians with interquartile ranges to describe continuous variables.

To report in‐hospital complications and outcomes, we excluded patients discharged alive within 24 hours. To report treatments, procedures, and tests, we excluded patients who had an LOS of 24 hours or shorter. We further excluded patients who died or withdrew care during hospitalization for the analysis of medications at discharge.

Statistical analysis was performed with SAS software (version 9.3; SAS Institute Inc., Cary, NC). Plots were made by R software (version 3.3.3; R Foundation for Statistical Computing, Vienna, Austria).

## Results

**Figure 1:**
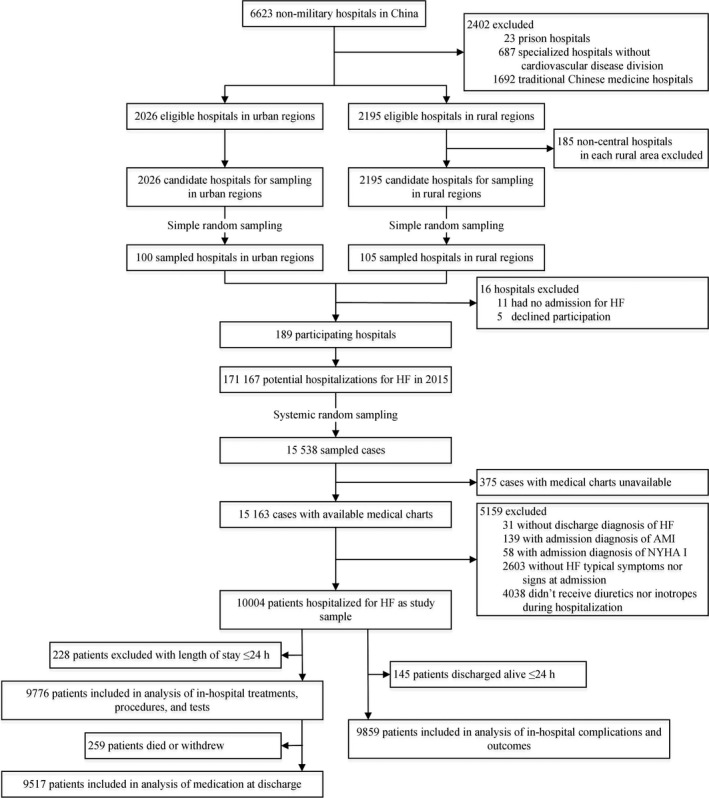
Study profile. AMI indicates acute myocardial infarction; HF, heart failure; NYHA, New York Heart Association.

According to the Ministry of Health, China had 6623 nonmilitary hospitals in 2011 (Figure 1). We excluded 23 prison hospitals, 687 specialized hospitals without divisions for cardiovascular disease, and 1692 traditional Chinese medicine hospitals. The sampling framework comprised 2010 central hospitals in 2010 rural regions in 3 rural strata and 2026 tertiary and secondary hospitals in 287 urban regions in 2 urban strata. We sampled 205 hospitals and invited them to participate in the study. We excluded 11 hospitals because they did not admit patients with HF and 5 declined to participate (including 2 tertiary hospitals in the Eastern urban and 3 tertiary hospitals in Central‐western urban regions). Examination of patient databases from the 189 remaining hospitals yielded 171 167 hospital admissions with discharge diagnosis of HF in 2015. We sampled 15 538 cases and acquired medical records for 15 163 (97.6%) of these cases. We subsequently excluded 5159 cases that did not meet the study criteria to create the study sample of 10 004 patients hospitalized for HF. Among the final study sample of 10 004 patients, 7012 met all 3 criteria for inclusion: symptoms and signs of HF and in‐hospital use of diuretics.

### Demographics

The median age of the cohort was 73 years (interquartile range, 65–80), and 48.9% were women (Table 1). More than half (56.2%) of the patients were hospitalized in rural areas. In stratified analysis, patients of non‐Han ethnicity were a larger proportion of the population hospitalized in rural centers compared with urban hospitals (14.5% versus 2.9%).

**Table 1:** Baseline Characteristics of Patients Hospitalized With HF in China

	Overall (n=10 004)
Social demographic	
Age (y), median, IQR	73 (65, 80)
Female, %	48.9
Han, %	90.6
Past or current smoking, %	21.7
Alcohol or drug abuse, %	3.9
Medical history, %	
HF	29.6
Myocardial infarction	8.1
Pacemaker	1.5
ICD	0.1
CRT‐D	0.1
Dialysis	0.7
Comorbidities, %	
Cardiac	
Coronary artery disease	60.6
Hypertension	53.8
Atrial fibrillation	35.9
Atrial flutter	1.4
Cardiac valvular disease	33.5
Pericardial diseases	2.7
Noncardiac	
COPD or asthma	30.4
Dyslipidemia	50.4
Stroke/transient ischemic attack	20.1
Diabetes mellitus	19.9
Chronic renal insufficiency	16.5
Peripheral vascular disease	8.8
Cancer	3.6
Anemia	25.9

ACS indicates acute coronary syndrome; COPD, chronic obstructive pulmonary disease; CRT‐D, cardiac resynchronization therapy with defibrillator; HF, heart failure; ICD, implantable cardioverter defibrillator; IQR, interquartile range.

### Clinical Presentation

**Figure 2:**
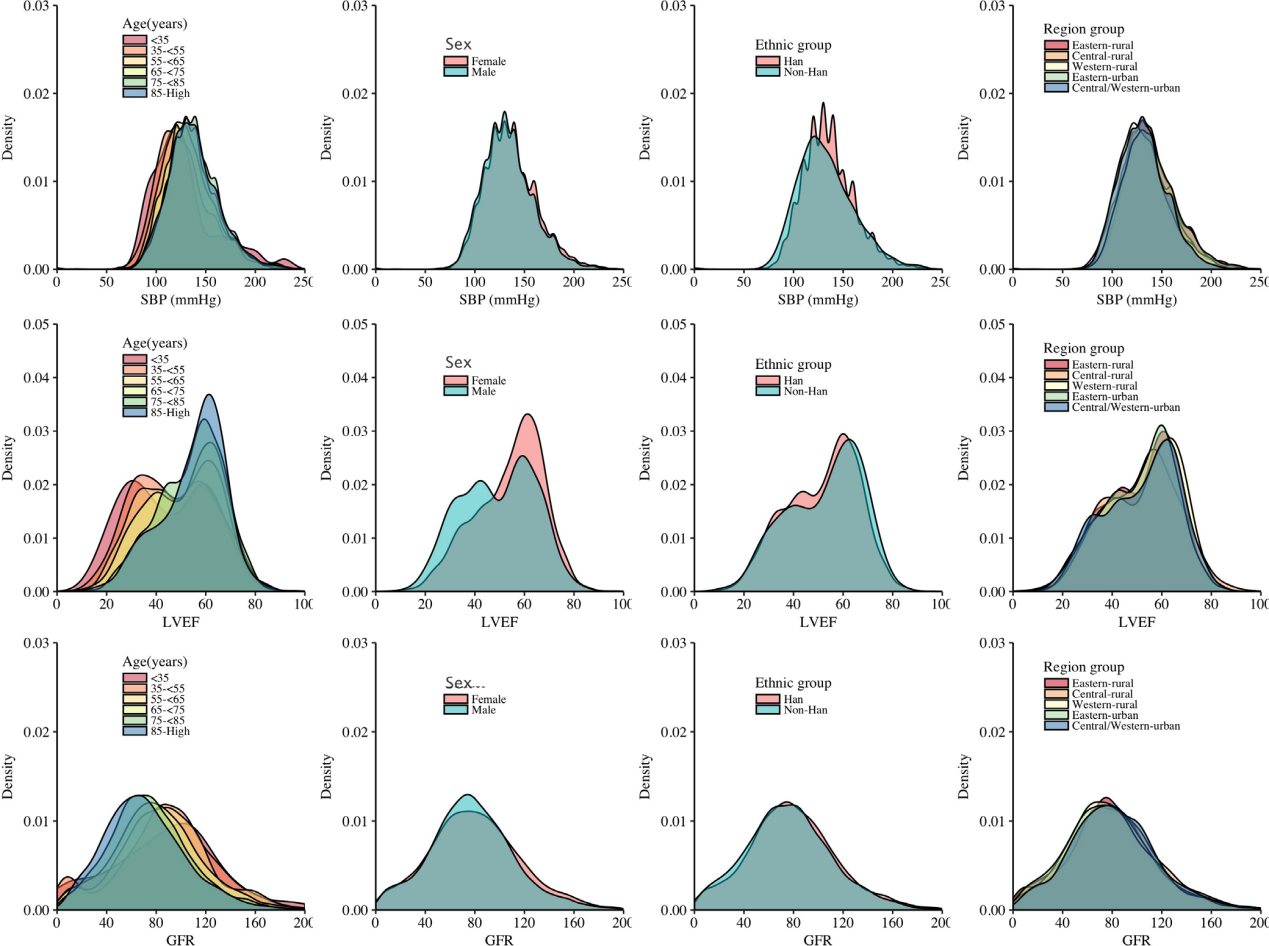
Density plots of systolic blood pressure at admission, ejection fraction, and glomerular filtration rate at admission in patients with HF in China. GFR indicates glomerular filtration rate; HF, heart failure; LVEF, left ventricular ejection fraction; SBP, systolic blood pressure.

Dyspnea at rest (51.4%) and exertion (50.0%) were the most frequently reported HF symptoms, and pulmonary rales (55.6%) and lower extremity edema (50.1%) were the most frequently reported HF signs (Table 2). These symptoms were observed with similar frequency when stratified by ejection fraction groups as well (Table S2). The majority of patients reported chest pain or discomfort at presentation (64.6%). A high prevalence of comorbidities was noted among these patients, and prevalence of coronary artery disease, hypertension, and dyslipidemia was 60.6%, 53.8%, and 50.4%, respectively. Only 29.6% had documented previous history of HF. Previous history of myocardial infarction, HF, smoking, and comorbidities of coronary artery disease, atrial arrhythmias, and valvular disease were more prevalent in patients hospitalized in urban areas compared with rural areas. Patients with New York Heart Association class III and IV comprised 40.1% and 30.9% of patients hospitalized with HF, respectively. Only 0.2% of patients presented with cardiogenic shock. Distribution of systolic blood pressure and glomerular filtration rate was similar among different age, sex, ethnicity, and region groups (Figure 2). Among the 63.6% of patients who underwent echocardiography during hospitalization, prevalence of LVEF ≥50%, 40% to 50%, and <40% was 60.3%, 17.7% and 22.0%, respectively.

**Table 2:** Clinical Presentation and Laboratory Tests for Patients Hospitalized with HF in China

	Overall (n=10 201)
Clinical presentation	
Dyspnea at rest, %	51.4
Orthopnea, %	27.2
Dyspnea on exertion, %	50.0
Paroxysmal nocturnal dyspnea, %	15.4
Fatigue, %	20.1
Edema, %	37.8
Chest pain, %	64.6
Cardiogenic shock, %	0.2
Heart rate (bpm), median, IQR	88 (76, 104)
Systolic blood pressure (mm Hg), median, IQR	130 (120, 150)
Diastolic blood pressure (mm Hg), median, IQR	80 (70, 90)
Body weight (kg), median, IQR	60 (52, 70)
Jugular vein distension, %	26.1
S3 present, %	0.2
Pulmonary rales, %	55.6
Hepatojugular reflux positive, %	5.4
Lower extremity edema, %	50.1
NYHA functional class, %	
II	10.9
III	40.1
IV	30.9
Unrecorded	18.1
GWTG‐HF risk score, median, IQR	36.0 (32.0, 41.0)
Admission to ICU, %	5.7
Laboratory tests (median, IQR)	
Blood urea nitrogen, mmol/L	6.9 (5.2, 9.7)
Serum creatinine, μmol/L	84.4 (67.0, 110.3)
Serum sodium, mEq/L	139.5 (136.4, 142.0)
Serum potassium, mmol/L	4.0 (3.6, 4.4)
LDL‐C, mmol/L	2.3 (1.8, 3.0)
Glucose, mmol/L	6.1 (5.1, 7.7)
Hemoglobin, g/L	129.0 (113.0, 143.0)
BNP	1321 (404, 3512)
NT‐pro‐BNP	2614 (993, 5530)

BNP indicates B‐type natriuretic peptide; GWTG‐HF, Get With the Guidelines‐Heart Failure; HF, heart failure; ICU, intensive care unit; IQR, interquartile range; LDL‐C, low‐density lipoprotein cholesterol; NT‐pro‐BNP, N‐terminal pro–B‐type brain natriuretic peptide; NYHA, New York Heart Association.

### Management

B‐type natriuretic peptide or N‐terminal pro–B‐type brain natriuretic peptide testing was performed in more than half (56.4%) of the patients. Chest imaging, including chest x‐rays or computed tomography scans, was obtained in 75.2% of the patients (53.5% for chest x‐ray and 35.5% for chest computed tomography scan). ECG was obtained in almost all patients (95.3%). Echocardiogram was performed in 63.6% of patients during hospitalization.

**Figure 3:**
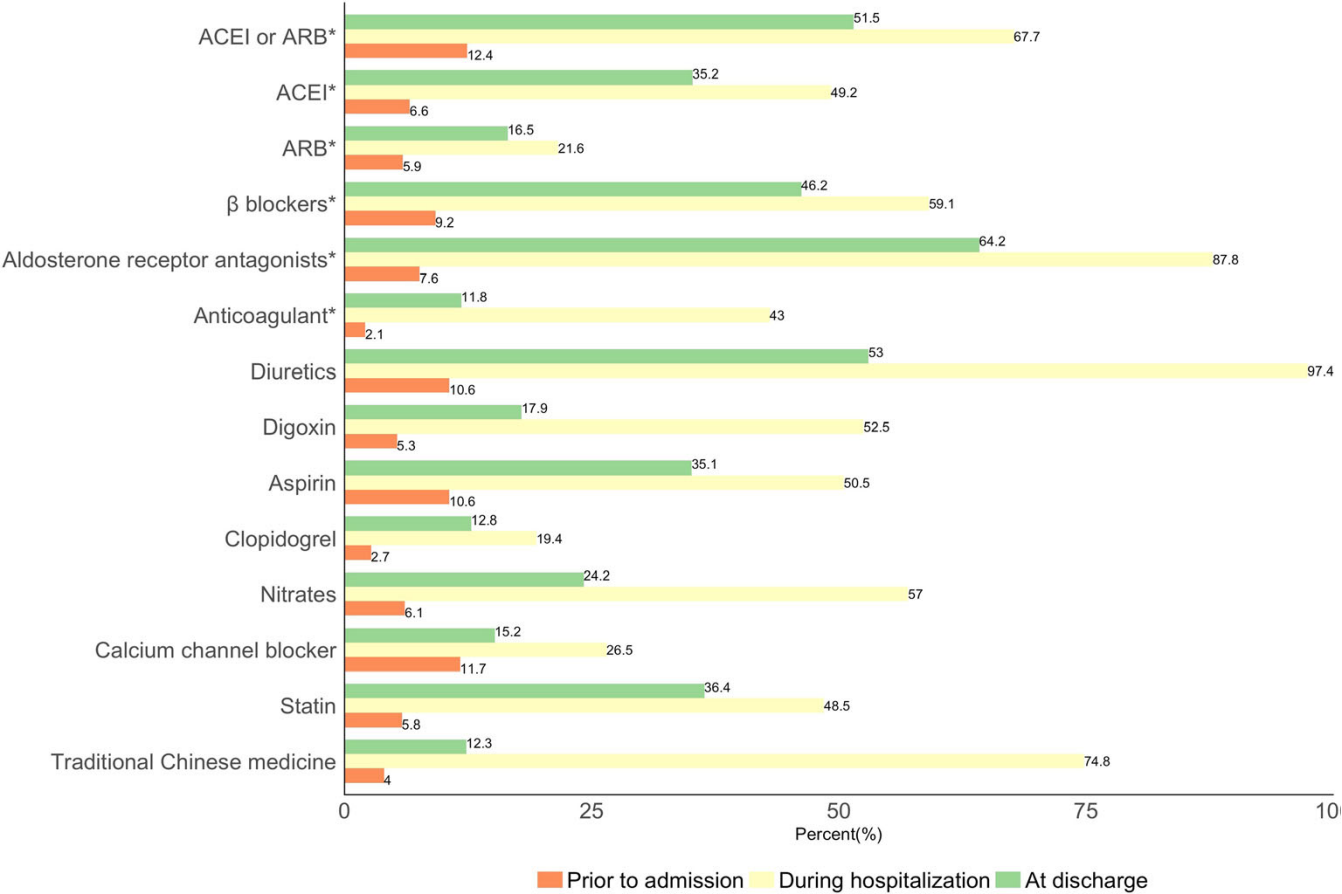
Medications before admission, during hospitalization, and at discharge for patients with HF in China. ACEI indicates angiotensin‐converting enzyme inhibitor; ARB, angiotensin receptor blocker; HF, heart failure. *Analysis for medications prescribed during hospitalization and at discharge was among patients with indications and without contraindications.

Approximately 6.6%, 5.9%, and 9.2% of patients were taking ACEIs, ARBs, and beta‐blockers, respectively, before admission. Overall, ACEIs, ARBs, beta‐blockers, and aldosterone receptor antagonists were administered to 32.7%, 21.5%, 44.9%, and 72.8% of patients at some point during hospitalization, respectively. Among ideal candidates with HF with reduced ejection fraction (ejection fraction, <40%), utilization rates of ACEIs and ARBs were 49.2% and 21.6%, respectively, and 67.7% for either during hospitalization (Figure 3). At discharge, prescription rates of ACEIs and ARBs were 35.2% and 16.5%, respectively, and 51.5% for either ACEIs or ARBs. Among eligible candidates with HF with reduced ejection fraction, prescription rates of beta‐blockers and aldosterone receptor antagonists were 59.1% and 87.8% during hospitalization and 46.2% and 64.2% at discharge, respectively. For eligible patients with atrial fibrillation, anticoagulants were administered to 43.0% of patients during hospitalization and prescribed to 11.8% patients at discharge.

Around 74.8% of the patients received traditional Chinese medications during hospitalization, including 69.1% who received intravenous medications. Rates of utilization of implantable cardioverter defibrillators (0.1%) or cardiac resynchronization therapy (0.04%) were low during hospitalization. Follow‐up appointments were documented at discharge for 24.3% of the patients.

### Outcomes

**Figure 4:**
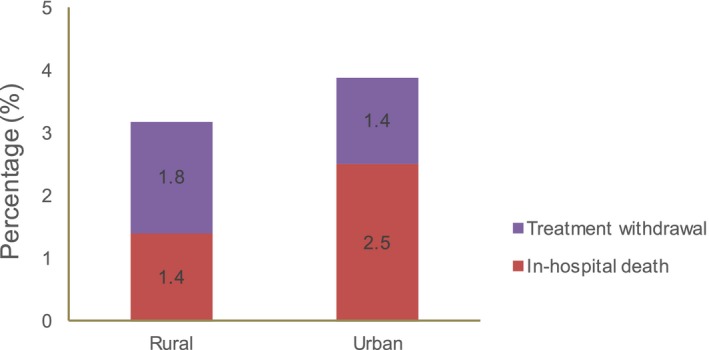
In‐hospital death and treatment withdrawal for HF in rural and urban areas in China. HF indicates heart failure.

Details of in‐hospital complications are provided in Table 3. Bleeding occurred in 2.6% of patients. Median hospital LOS was 9 days (interquartile range, 7–13). Overall, 1.9% patients died and care was terminally withdrawn for another 1.6%. The combined rate of in‐hospital death and treatment withdrawal was 3.2% in rural areas and 3.9% in urban areas (Figure 4).

**Table 3:** In‐Hospital Complications and Outcomes of Patients Hospitalized With HF in China

	Overall (n=10 052)
Length of stay (days) median, IQR	9 (7, 13)
Complications, %	
Bleeding	2.6
Cardiogenic shock	1.2
DVT or PE	0.5
Stroke	0.3
Myocardial infarction	0.1
Mortality, %	
Death	1.9
Treatment withdrawal	1.6

DVT indicates deep venous thrombosis; HF, heart failure; IQR, interquartile range; PE, pulmonary embolism.

## Discussion

In the contemporary era, patients admitted with acute decompensated HF in China are, on average, in their seventies, and few had known preexisting HF. We identified substantial gaps in care, including underutilization of diagnostic tests such as echocardiograms, chest imaging, and biomarker testing, low prescription rates of guideline‐recommended medications, suboptimal rates of follow‐up appointments, and widespread utilization of therapies of unknown effectiveness like traditional Chinese medicine. In addition, these patients suffered relatively low rates of complications and inpatient mortality, suggesting hospitalization of low‐acuity patients.

This study extends the earlier literature by providing a contemporary national landscape of HF care in China. Compared with previous studies from China, patients in our study were older with higher prevalence of HF with preserved ejection fraction, were prescribed evidence‐based HF medications much less frequently, and experienced lower mortality rates. Our study, in contrast to previous studies, is nationally representative, which may explain the differences. Most of the earlier studies were either limited by size or scope or conducted primarily in urban or tertiary care settings.^12^, ^19^, ^20^, ^21^, ^22^, ^23^ Rural populations, accounting for around half of the Chinese population,^6^ have different characteristics compared with urban populations. Patients hospitalized in rural areas in our study more frequently included minority ethnicities, had significantly lower burden of comorbidities, and had lower rates of in‐hospital mortality compared with those admitted to urban hospitals.

We observed some notable characteristics among Chinese patients hospitalized with HF compared with their counterparts in other countries from published international registries (Table 4). First, their average age was 73, 8 years higher than that observed more than a decade ago.^24^ Whereas a difference in sampling strategies could partially account for this discordance, it is also likely that increasing longevity of the Chinese population has led to this trend, mirroring what has been observed globally.^25^, ^26^ Second, among all patients admitted for HF, only approximately one‐third reported a previous history of HF, and only one‐third of these were on any ACEIs, ARBs, or beta‐blockers or aldosterone antagonists before hospitalization. The rate of preexisting diagnosis of HF at the time of hospitalization is much lower than around 60% reported in the United States^27^ and Europe.^26^ Whereas it is possible that a significant proportion of these patients developed new HF corroborating increasing prevalence in an epidemiological transition, or that documentation in the admission records was incomplete, it is also likely that a lot of patients remained undiagnosed, particularly in the rural setting. In 2003, a study in China involving self‐reporting of HF by a random sample of 15 518 individuals suggested underdiagnosis of HF in rural areas.^1^ Third, among patients in whom LVEF was measured, 60% had HF with preserved ejection fraction, a higher prevalence than that documented from inpatient registries in the United States^27^ and other countries.^26^, ^28^ Although this is in alignment with the global trend of increasing HF with preserved ejection fraction prevalence, secondary to increasing recognition and changing epidemiology of HF,^27^ nonspecific diagnostic criteria for HF with preserved ejection fraction also render it prone to misdiagnosis.^29^ Fourth, at presentation, around 70% of patients had New York Heart Association class III/IV HF, and cardiogenic shock was documented in only 0.2% of patients, much lower estimates compared with other countries.^30^, ^31^ These disparities possibly suggest lower thresholds for inpatient admission leading to hospitalization of low‐acuity patients in China.

**Table 4:** Comparison of China PEACE 5r‐HF With Other International Registries

	China PEACE	KorAHF^42^	ATTEND^31^	GWTG‐HF^25^	EHFS II^26^	Gulf CARE^28^	THESUS‐HF^41^
Region	China	Korea	Japan	US	Europe	Middle East	Africa
Time period	2015	2011 to 2012	2007 to 2011	2005 to 2010	2004 to 2005	2012	2007 to 2010
Sample size	10 004	2066	4842	110 621	3580	5005	1006
Demographics							
Age (SD/Q or median/IQR), y	73 (65, 80)	69 (±14)	73 (±14)	74 (62, 83)	70 (±12.5)	59 (±15)	55 (39, 67)
Male, %	51.1	59	58	53	61	63	49
Past or current smoking, %	21.7	N/A	42.5	18	N/A	22	9.8
Medical history							
Previous HF	29.9	N/A	N/A	58	62.9	N/A	N/A
Comorbidities, %							
Coronary artery disease	60.6	N/A	N/A	50	N/A	47	N/A
Hypertension	53.8	59	69.4	76	N/A	61	55
Atrial fibrillation	35.9	27	36	31	N/A	12	18.3
COPD or asthma	30.4	11	9.5	30	N/A	N/A	N/A
Dyslipidemia	50.4	N/A	36.6	44	N/A	36	9.2
Stroke/transient ischemic attack	20.1	N/A	14	14	N/A	8.1	N/A
Diabetes mellitus	19.9	36	33.8	43	N/A	50	11.4
Chronic renal insufficiency	16.5	N/A	N/A	50	N/A	15	7.7
Clinical presentation							
Cardiogenic shock	0.2	N/A	N/A	N/A	4	N/A	8
Heart rate, bpm	88 (76, 104)	91 (26)	99 (29)	82 (70, 98)	N/A	97 (23)	104 (90, 116)
Systolic blood pressure, mm Hg	130 (120, 150)	136 (31)	146 (37)	138 (118, 159)	N/A	137 (34)	127 (106, 150)
NYHA class III to IV, %	71.0	N/A	81.4	>90	N/A	75	34.6
EF<40% (%)	22.0 among EF measured	56	53.4	50	66 for EF<45%	69	>50
Medication before admission, %							
ACEI	6.6	12	14	N/A	55	43	75 for ACEI/ARB
ARB	5.9	27	33	N/A	9	13	
Beta‐blocker	9.2	27	32	N/A	43	44	18
Medication at discharge, %							
Diuretic	53.0	N/A	83	N/A	90.1	94	80 for loop diuretics
ACEI or ARB^a^	51.5	N/A	N/A	92	72 for ACEI, 10 for ARB	61 for ACEI, 17 for ARB	81
Beta‐blocker^a^	46.2	44	68	94	61	71	31
Aldosterone receptor antagonists^a^	64.2	40	50	28	48	43	77
Anticoagulant^a^	11.8	N/A	44 for warfarin	82	33 for warfarin	19	20
Digoxin	17.9	N/A	16	N/A	31	25	62
Tests							
Echocardiogram	63.6	91	N/A	99	86	91	N/A
Chest x‐ray or CT scan	75.2	N/A	N/A	N/A	≈100	N/A	N/A
Procedures, %							
Mechanical ventilation	2.8	13.6	7.5	N/A	5	8.5	N/A
Pacemaker	0.3	N/A	3.8	N/A	2.7	N/A	N/A
ICD	0.1	1.4	2.6	5.4 among HFrEF	1.2	1.1	N/A
CRT	0.04	1.3	2.3	6.7 among eligible patients	N/A	0.6	N/A
PCI	0.9	10	8.0	1	8.4	6.0	N/A
CAG	3.0	N/A	N/A	10	N/A	N/A	N/A
Outcomes							
Length of stay (median), d	9 (7, 13)	8	21	4	9 (6, 14)	7 (3, 10)	7
Mortality, %	3.5 when plus treatment withdrawal	6.1	6.4	3	6.7	6.3	4.2

ACEI indicates angiotensin‐converting enzyme inhibitor; ARB, angiotensin receptor blocker; ATTEND, Acute Decompensated. Heart Failure Syndromes registry; CAG, coronary angiography; China PEACE, Patient‐centered Evaluative Assessment of Cardiac Events; COPD, chronic obstructive pulmonary disease; CRT, cardiac resynchronization therapy; CT, computed tomography; EF, ejection fraction; EHFS II, EuroHeart Failure Survey II; Gulf CARE, Gulf Acute Heart Failure Registry; GWTG‐HF, Get With the Guidelines‐Heart Failure; HF, heart failure; HFrEF, heart failure with reduced ejection fraction; ICD, implantable cardioverter defibrillator; IQR, interquartile range; KorAHF, Korean Acute Heart Failure registry; N/A, not applicable; NYHA, New York Heart Association; PCI, percutaneous coronary intervention; THESUS‐HF, The Sub‐Saharan Africa Survey of Heart Failure.

a Analysis among patients with indications and without contraindications.

Moreover, we noted differences in epidemiology and outcomes among Chinese patients with HF in our study compared with the China‐HF (China Heart Failure) study (Table S3). We believe that differences in study design, sampling strategies, and inclusion/exclusion criteria between the 2 studies likely explain the differences in findings. The China‐HF registry was a prospective study in which patients had to have imaging evidence of chest congestion or structural heart disease in addition to signs/symptoms to be included in their study. As such, these criteria are more specific, albeit less sensitive, given that patients who did not receive chest imaging or an echocardiogram were not included in their study. In contrast, our study is retrospective and did not require patients to have had chest imaging or an echocardiogram during their hospitalization. Our inclusion criteria relied on presence of signs/symptoms of HF and in‐hospital treatment with diuretics or inotropes in addition to *ICD‐10* codes or principal discharge diagnosis terms for HF. Around 38% patients included in our study did not receive an echocardiogram, 25% did not receive chest imaging, and 15.7% did not receive either. As such, we believe that our criteria are more sensitive and adequately specific (though less specific than China‐HF) for patients admitted with HF exacerbation in the real‐world setting. Moreover, they did not exclude patients who had AMI, and these comprised around 14% of patients included in their study. We excluded patients with AMI in our study, contributing to the differences in epidemiology between the 2 studies. Patients with AMI at admission resulting in HF would more likely cause reduced ejection fraction that might explain higher prevalence of HF with reduced ejection fraction noted in the China‐HF study compared with our study—China PEACE 5r‐HF.

We also identify substantial gaps in HF care in China. An echocardiogram was not performed in 1 of 3 patients admitted with HF in our study despite strong guideline support and the importance of LVEF to guiding HF therapy.^9^, ^32^ This is much worse than estimates from the United States^33^ and the Europe,^26^ where <15% of patients do not receive an echocardiogram. Furthermore, guideline‐recommended therapies were substantially underutilized. Among ideal patients, ACEIs/ARBs and beta‐blockers were prescribed in only half of the patients, and anticoagulation was prescribed in only 1 in 10 eligible patients at discharge. This finding has important prognostic implications given that in‐hospital initiation of therapies is one of the best predictors of their long‐term use.^34^, ^35^ These numbers contrast with those in the United States, where prescription rates of ACEIs/ARBs and beta‐blockers now exceed 90% and anticoagulation exceeds 80%.^33^ It is noteworthy that adherence in the United States was much lower before the development of registries like Get With the Guidelines (GWTG)^36^ and the Acute Decompensated Heart Failure National Registry (ADHERE)^37^ and subsequent policy initiatives. There is scope for similar efforts by policy makers in China to incentivize improved prescribing of appropriate medications to suitable patients with HF.

We also note that approximately 3 of 4 patients received traditional Chinese medicine during hospitalization.^38^ Traditional Chinese medicine is practiced as a complementary approach for cardiovascular disease. Currently available randomized controlled trials on traditional Chinese medicine are generally small in size and have shown equivocal benefit, making it difficult to draw definite conclusions about their efficacy and safety.^39^ Further research is needed to elucidate the clinical benefit of traditional Chinese medicine for the management of HF.

The combined rate of in‐hospital mortality and treatment withdrawal in our study was around 3.4%,^24^ only slightly higher than the United States.^25^ Corresponding estimates in other countries, however, have ranged from 4% to 13%.^26^, ^28^, ^31^, ^40^, ^41^, ^42^, ^43^, ^44^ Relatively better in‐hospital outcomes in China are likely secondary to lower acuity of patients hospitalized with HF when compared with Western nations. This is even more likely given the massive divide in out‐ and inpatient insurance coverage in China, making it easier for patients with HF to obtain necessary medical care in the inpatient setting.^45^, ^46^ Poor rates of outpatient insurance coverage (<50%) and almost universal inpatient coverage (>70%) may incentivize hospitalization of low‐acuity patients.^46^ Moreover, mortality rates were higher in urban areas compared with rural areas. A similar gap has been observed for mortality rates in urban versus rural areas for AMI in China.^47^ Higher mortality rates in urban areas reflect the sicker profile of patients admitted to their hospitals or poorer inpatient therapy. In addition, it is noteworthy that the median LOS of patients hospitalized for acute decompensated HF was more than double compared with that in the United States (9 versus 4 days). Relatively low rates of in‐hospital complications and mortality, despite a much longer median LOS in China, suggest admission of low‐acuity patients and subsequent excessive utilization of healthcare resources, providing opportunities for optimizing efficiency of HF care in China.

The findings of this study should be interpreted in view of several limitations. First, we measured clinical characteristics on the basis of documentation in medical records. Definitions of some disorders and completeness of documentation can differ across hospitals. Second, we did not include death before or after discharge as an outcome. We could only measure in‐hospital outcomes, because we were unable to link patient‐level data to a national registry of deaths. Finally, LVEF was not assessed in 1 of 3 of the patients. As such, we could not accurately estimate the proportion of patients with HF with reduced ejection fraction and utilization of guideline‐recommended therapies among them. This is, however, an important finding given that evaluation of LVEF in patients admitted with acute HF is 1 of the quality indicators for HF care.

We conclude that patients admitted with acute HF in China have distinctive epidemiology, receive substandard care, and have lower inpatient mortality. We demonstrated substantial gaps between real‐life practice and guideline‐recommended care for these patients. The reasons for relatively lower rates of inpatient mortality despite longer LOS are unclear, but could likely be attributed to hospitalization of patients with HF who could otherwise be treated in the outpatient setting. Our findings underscore the need for national initiatives to further understand the reasons for existing gaps in care and implement tools and strategies to mitigate these factors and improve adoption of evidence‐based care for HF in China.

## Author Contributions

Drs Krumholz and Li designed the study and take responsibility for all aspects of it. Drs Yu and Gupta wrote the first draft of the article, with further contributions from Drs Masoudi, Du, Zhang, Krumholz and Li. Dr Wu performed statistical analysis. All authors approved the final version of the article.

## Sources of Funding

This project was supported by the National Key Technology R&D Program (2015BAI12B02, 2017YFC1310801, and 2017YFC1310803) from the Ministry of Science and Technology of China, CAMS Innovation Fund for Medical Sciences (CIFMS 2016‐I2M‐2‐004), and the 111 Project from the Ministry of Education of China (B16005). Dr Gupta is supported by grant T32 HL007854 from the National Heart, Lung and Blood Institute of the National Institutes of Health. The Chinese government funded the study and had no role in study design, data collection, data analysis, data interpretation, or writing of the report. The corresponding author had full access to all the data in the study and had final responsibility for the decision to submit for publication.

## Disclosures

Dr Krumholz was a recipient of a research grant, through Yale, from Medtronic and the US Food and Drug Administration to develop methods for postmarket surveillance of medical devices; was a recipient of a research grant with Medtronic and Johnson & Johnson, through Yale, to develop methods of clinical trial data sharing; was a recipient of a research agreement, through Yale, from the Shenzhen Center for Health Information for work to advance intelligent disease prevention and health promotion; collaborates with the National Center for Cardiovascular Diseases in Beijing; chairs a Cardiac Scientific Advisory Board for UnitedHealth; is a participant/participant representative of the IBM Watson Health Life Sciences Board; is a member of the Advisory Board for Element Science, the Advisory Board for Facebook, and the Physician Advisory Board for Aetna; and is the founder of Hugohealth, a personal health information platform. Dr Masoudi has a contract with the American College of Cardiology for his role as Chief Science Officer of the NCDR. Dr Gupta is a member of Heartbeat Health, Inc, a preventive cardiology platform. Drs Krumholz and Gupta received payment from the Arnold & Porter Law Firm for work related to the Sanofi clopidogrel litigation and from the Ben C. Martin Law Firm for work related to the Cook inferior vena cava filter litigation. The remaining authors have no disclosures to report.

## Supporting information


**Appendix S1.** China PEACE 5r‐HF Study Site Investigators by Hospital.
**Data S1.** The 8 Categories of Traditional Chinese Medicines Commonly Used in China Among Patients With Heart Failure.
**Data S2.** Definitions of In‐Hospital Complications as Provided in the Case‐Abstraction Forms.
**Table S1.** Definition for Eligible Patients
**Table S2.** HF‐Specified Signs/Symptoms and Diuretic Use Stratified by EF Groups
**Table S3.** Comparison of China PEACE 5r‐HF With China‐HF Study
