# Supplementary material for: Characteristics, Management, and Outcomes of Patients Hospitalized for Heart Failure in China: The China PEACE Retrospective Heart Failure Study
Source: J Am Heart Assoc. 2019 Aug 21;8(17):e012884. doi: 10.1161/JAHA.119.012884 (PMC6755852; doi:10.1161/JAHA.119.012884)
Supplement: Supplementary file 1 — Appendix S1. China PEACE 5r‐HF Study Site Investigators by Hospital. Data S1. The 8 Categories of Traditional Chinese Medicines Commonly Used in China Among Patients With Heart Failure. Data S2. Definitions of In‐Hospital Complications as Provided in the Case‐Abstraction Forms. Table S1. Definition for Eligible Patients Table S2. HF‐Specified Signs/Symptoms and Diuretic Use Stratified by EF Groups Table S3. Comparison of China PEACE 5r‐HF With China‐HF Study [file JAH3-8-e012884-s001.pdf]

SUPPLEMENTAL MATERIAL

TABLE OF CONTENTS

Table S1 Definition for eligible patients ... 3
Table S2 HF-specified signs/symptoms and diuretic use stratified by EF groups ... 3
Table S3 Comparison of China PEACE 5r-HF with China-HF study ... 5
Data S1 The eight categories of traditional Chinese medicines commonly used in China among patients with heart failure ... 7
Data S2 Definitions of in-hospital complications as provided in the case-abstraction forms ... 8
Data S3 China PEACE 5r-HF Study site investigators by hospital ... 10

Table S1 Definition for eligible patients

Eligible patients were defined as patients with indication and without contraindications for the following medications:

Medications	Indications	Contraindications
Angiotensin-converting-enzyme inhibitors (ACEI) or angiotensin receptor blockers (ARB)	Left ventricular ejection fraction (LVEF) < 40%	Allergy to ACEI/ARB<br>Hyperkalemia (serum potassium >5.5 mmol/L)<br>Creatinine >2.5 mg/dL in men or >2.0 mg/dL in women<br>Systolic blood pressure <90mmHg<br>Pregnancy<br>Moderate-to-severe aortic stenosis<br>Other documented contraindications
β blockers	LVEF < 40%	Allergy to β blockers<br>Asthma<br>Second or third degree atrioventricular block with no pacemaker implanted<br>Systolic blood pressure <90mmHg<br>Bradycardia (heart rate <55 beats/min) without taking a β blocker<br>Other documented contraindications
Aldosterone receptor antagonist	LVEF⩽35% and NYHA II-IV	Allergy to aldosterone receptor antagonist<br>Hyperkalemia (serum potassium >5.5 mmol/L)<br>Creatinine >2.5 mg/dL in men or >2.0 mg/dL in women<br>Systolic blood pressure <90mmHg<br>Combination of an ACEI and ARB<br>Other documented contraindications
Anticoagulants	Atrial fibrillation	Allergy to anticoagulants<br>Risk of bleeding or active bleeding<br>Other documented contraindications

Table S2 HF-specified signs/symptoms and diuretic use stratified by EF groups

%	**EF<40%**	**EF≥40%，<50**	**EF≥50%**	**No LVEF**

							Assessment	
	N	%	N	%	N	%	N	%
	1385	100	1117	100	3792	100	3710	100
Dyspnea at rest	723	52.2	579	51.8	1866	49.2	1974	53.2
Orthopnea	521	37.6	370	33.1	866	22.8	967	26.1
Dyspnea on exertion	728	52.6	579	51.8	1933	51.0	1760	47.4
Paroxysmal nocturnal dyspnea	312	22.5	217	19.4	543	14.3	467	12.6
Edema	503	36.3	407	36.4	1462	38.6	1406	37.9
Jugular vein distension	401	29.0	259	23.2	898	23.7	1057	28.5
S3 present	4	0.3	5	0.4	2	0.1	5	0.1
Pulmonary rales	738	53.3	608	54.4	1906	50.3	2312	62.3
Hepatojugular reflux positive	108	7.8	56	5.0	195	5.1	179	4.8
Lower extremity edema	652	47.1	532	47.6	1947	51.3	1879	50.6
Diuretic use	1374	99.2	1103	98.7	3683	97.1	3574	96.3

Table S3 Comparison of China PEACE 5r-HF with China-HF study

	China PEACE	China-HF[1]
Region	National rural and urban	National
Time period	2015	2012-2015
Sample size	10,004	13,687
Number of participating hospitals	189	132
**Demographics**		
Age (SD/Q), years	73 (65-80)	64.8 (15)
Male (%)	51.1	59.1
**Medical history**		
Prior HF	29.6	45.5
**Comorbidities (%)**		
Coronary artery disease	60.6	49.6
Hypertension	53.8	50.9
Atrial fibrillation	35.9	24.4
Valvular heart disease	33.5	15.5
Diabetes mellitus	20	21
Chronic renal insufficiency	16.5	46.7
**Clinical presentation (%)**		
NYHA class III-IV	70.0	76
EF<40% among EF measured	22.0	36.5
**Tests (%)**		
Echocardiogram	63.6	82.5
**Medication at discharge (%)**		
ACEI or ARB*	51.5	67.4
Beta-blocker*	46.2	70
Aldosterone receptor antagonists*	64.2	74.1
Anticoagulant*	11.8	16.1 for warfarin
**Outcomes**		
Length of stay (median), days	9 (7-13)	10 (7-15)
In-hospital mortality (%)	3.5 when plus treatment withdrawal	4.1

* Medications used among eligible patients
† Medications used during hospitalization among eligible patients
NYHA indicates New York Heart Association; EF, ejection fraction; ACEI indicates

angiotensin-converting enzyme inhibitor; ARB, angiotensin receptor blocker

Data S1 The eight categories of traditional Chinese medicines commonly used in China among patients with heart failure

(1) Salviamiltiorrhiza/ RedGinseng/ Ginseng (e.g. Danshendripping pills,Tanshinone)*
(2) Gingko (e.g. Ginkgobiloba, Ginkgobilobaextract)*
(3) Panax notoginseng (e.g. Panaxnotoginsengsaponins, Xueshuantongin jection)*
(4) Hirudin (e.g.Lepirudin, Shuxuetong injection)*
(5) Erigeron Breviscapus (e.g. Erigeronbreviscapus injection, Breviscapinun)*
(6) Astragalus (e.g. Qishenyiqi Dripping Pills, Qiliqiangxin capsule)*
(7) Lipid lowing agents (e.g. Xuezhikang, Taizhian)
(8) Other (e.g. Puerarin, Suxiaojiuxin pills, Kyushinpills)

* based on the main functional ingredient.

Data S2 Definitions of in-hospital complications as provided in the case-abstraction forms

1) Deep venous thrombosis (DVT) or pulmonary embolism (PE)

Indicate evidence of DVT or PE was documented in the medical record, referring to the in-hospital development of DVT or PE. Pre-existing DVT or PE prior to admission should not be counted. The documentation of DVT or PE must be confirmed by ultrasound, venous imaging or appropriate diagnostic modality.

2) Myocardial infarction

Indicate if there is physician documentation of myocardial infarction during hospitalization. A myocardial infarction is evidenced by any of the following:

1. A rise and fall of cardiac biomarkers (preferably troponin) with at least one of the values in the abnormal range for that laboratory [typically above the 99th percentile of the upper reference limit (URL) for normal subjects] together with at least one of the following manifestations of myocardial ischemia:

   a. Ischemic symptoms.

   b. ECG changes indicative of new ischemia (new ST-T changes, new left bundle branch block, or loss of R wave voltage).

   c. Development of pathological Q- waves in 2 or more contiguous leads in the ECG (or equivalent findings for true posterior MI).

   d. Imaging evidence of new loss of viable myocardium or new regional wall motion abnormality.

   e. Documentation in the medical record of the diagnosis of acute myocardial infarction based on the cardiac biomarker pattern in the absence of any items enumerated in a-d due to conditions that may mask their appearance (e.g., peri-operative infarct when the patient cannot report ischemic symptoms; baseline left bundle branch block or ventricular pacing).

2. Imaging evidence of a region with new loss of viable myocardium at rest in the absence of a non-ischemic cause. This can be manifest as:

   a. Echocardiographic, CT, MR, ventriculographic or nuclear imaging evidence of left ventricular thinning or scarring and failure to contract appropriately (i.e., hypokinesis, akinesis, or dyskinesis).

   b. Fixed (non-reversible) perfusion defects on nuclear radioisotope imaging (e.g., MIBI, thallium).

3. Medical record documentation of myocardial infarction.

3) Cardiogenic shock

Indicate if there is physician documentation of cardiogenic shock during hospitalization. Cardiogenic shock is defined as a sustained (>30 minutes) episode of systolic blood pressure <90 mm Hg, and/or cardiac index <2.2 L/min/m2 determined to be secondary to cardiac dysfunction, and/or the requirement for parenteral inotropic or vasopressor agents or mechanical support (e.g., intra-aortic balloon pump, extracorporeal circulation, ventricular assist devices) to maintain blood pressure and cardiac index above those specified levels.

4) Ischemic stroke

Indicate if there are physician documentations of new-onset ischemia stroke and stroke-related symptoms during hospitalization. The stroke-related symptoms include: trouble walking/loss of balance/incoordination, one-sided numbness or hemi-anesthesia, one-sided facial numbness or hemi-anesthesia, mouth askew and drooling, dysarthria or slurred speech, loss of vision or blurred version in one or both eyes, dizziness with vomiting, severe headache and vomiting, unconsciousness, and hyperspasmia.

5) Hemorrhagic stroke

Indicate if there are physician documentations of new-onset hemorrhagic stroke and stroke-related symptoms during hospitalization.

6) Bleeding

Indicate if the patient had a bleeding event during hospitalization. Bleeding is defined as documented bleeding event or the drop in hemoglobin of ≥3 g/dL

Data S3 China PEACE 5r-HF Study site investigators by hospital

Anhui Province, Dingyuan County General Hospital, Xinming Ma, Wenhua Zhang; Huangshan Third People's Hospital, Changjie Hong, Fang Wang; Mengcheng County First People's Hospital, Gaofeng Guo; Beijing, Peking University People's Hospital, Hong Chen, Huiping Li, Yu Luo; Beijing Watson Hospital, Lihua Shang, Jing Wang, Xinhua Xu; Yanqing District Hospital of Beijing, Li Yang, Xiaolei He; Chongqing, Tongliang County People's Hospital, Guofu Li; Chongqing Sixth People's Hospital, Yonghong Huang; Fuling Central Hospital of Chongqing, Liquan Xiang, Lin Ning, Peng Xiao; Nanchuan District People's Hospital of Chongqing, Lingxian Zeng; Fujian Province, Fujian Provincial Hospital, Yansong Guo, Lirong Lin; Longyan City, Fujian Province First Hospital, Haiming Yi, Kaihong Chen, Yong Lin; Wuyishan City, Fujian Province Hospital, Qingfei Lin, Chunxia Liu; Fuzhou First Affiliated Hospital of Fujian Medical University, Yan Zhang, Yu Ouyang, Chuanchuan Li; Nanan Hospital, Duanping Dai, Shaoxiong Hong; Zhouning County Hospital, Banghua He, Miaoli Huang; Gansu Province, Minxian County People's Hospital of Gansu Province, Yuhong Liu, Minwu Bao, Hongliang Chu; Jiuquan City People's Hospital, Yaofeng Yuan, Zhirong Li; Hongzhou District, Lanzhou City People's Hospital, Ping Zhang; Sunan Yugur Autonomous County People's Hospital, Zhansheng Ba, WanHai Fu; Zhangjiachuan Hui Autonomous County People's Hospital, Shitang Gao, Qiang Gao; Zhuoni County People's Hospital, Hong Li; Guangdong Province, Peking University Shenzhen Hospital, Chun Wu, Huan Qu, Yinlong Du; Panyu District, Guangzhou City Central Hospital, Guoqin Chen, Jinliang Li; Huizhou City People's Hospital, Yuansheng Shen, Lizhen He, Jian Shen; Qujiang District People's Hospital, Jianfan Chen, Jiajia Dai; Wuchuan City People's Hospital, Yuanming Yi, Xuelian Deng; Guangxi Province, Fengshan County People's Hospital, Wen Long, Shilin Lu, Jianhua Huang; Gongcheng Yao Autonomous County People's Hospital, Mingfang Feng, Mao Rong; Guilin People's Hospital, Diguang Pan,Wenhua Tang, Yi Ding; Guiping City People's Hospital, Guang Chen, Yongxian Rong; Jingxi County People's Hospital, Wen Liang, Xianfeng Liang; Laibin Xingbin Bayi Hospital, Chunhua He, Pudi Huang; Liujiang County People's Hospital, Meifa Wei, Pinyi Tan; Luchuan County People's Hospital, Min Feng, Guanhong Chi; Guizhou Province, Guiyang Medical College Hospital, Lirong Wu, Wei Li, Haijun Guo; Puding County People's Hospital, Wei Jiang, Chen Yuan; Rongjiang County People's Hospital, Fangning Wang; Shougang Shuicheng Iron and Steel (Group) Co., Ltd. General Hospital, Min Zhang, Lala Li; Hainan Province, Hainan West Central Hospital (Pizhou First People's Hospital), Zhongwei Wu; Hainan Medical College Hospital, Yueqiong Kong, Yang Yang; Hebei Province, Baoding Second Central Hospital, Guang Ma， Jing Zhao; Second Affiliated Hospital of Hebei North University, Wenhui Li, Peitian Han; First Affiliated Hospital of Hebei North University, Fangjiang Li, Aiai Zhang, Feixing Li; Jize County Hospital, Qiu'e Guo, Han Cui, Ruihong Li; Jingxing Hospital, Zhenhai Zhao, Jun Yin; Kangbao County People's Hospital, Ruiqing Zhao, Guangjun Song; Laoting County Hospital, Keyong Shang; Shijiazhuang City Luan City People's Hospital, Ruigang Zhao, Tao Jia; Kaiping Hospital of Tangshan City, Yanmin Yao, Yaoqi Liu; Wuqiang County People's Hospital, Binglu Liu, Hongguang Zhang; Henan Province, First Affiliated Hospital of Henan University of Science and Technology, Pingshuan Dong, Xuming Yang, Laijing Du; Henan Provincial People's Hospital, Chuanyu Gao, Xinyun Liu; Kaifeng Integrative Medicine Hospital, Lei Qin, Jieyun Liu, Yaoxin Wang; Luyi County People's Hospital, Yuanxun Xu, Anran Guo, Yangguang Zhao; Biyang County People's Hospital, Weijuan Zhou, Guoyuan Yin; Nanyang Central Hospital, Shouzhong Yang, Jianbu Gao; Qinyang City People's Hospital, Xiaowen Ma, Yanli Liang; Queshan County People's Hospital, Guoyin Fan, Fucheng Yuan, Yaoze Li; Ruyang County People's Hospital, Chengning Shen; Shangqiu Fourth People's Hospital, Jianjun Pan; Shangqiu Long March People's Hospital, Qian Wang, Min Li; Xinmi City First People's Hospital, Jie Dou, Xiao Wang; Xiuwu County People's Hospital, Jianbao Chang, Lihua Wu; Zhengzhou People's Hospital, Hengliang Liu, Lei Mao; Heilongjiang Province, Baiquan County People's Hospital, Yachen Zhang; Gannan County

People's Hospital, Mei Chen, Xin Jin, Xiaodong Li; Harbin 242 hospital, Jiubin Sun, Yin Zhou; Harbin Daoli District People's Hospital, Yongfan Jin, Tianwei Luan; Second Affiliated Hospital of Harbin Medical University, Bo Yu, Zhengqiu Wang, Yibo Guo; Hegang Mining Group Co., Ltd. General Hospital, Xiaowen Pan, Yaqin Liu; Jixi City People's Hospital, Jia Wang, Yan Xin; First Affiliated Hospital of Jiamusi University, Zhaofa He, Feng Zhang; Lindian County Hospital, Wenzhou Li, Dongyue Xu; Yichun Second People's Hospital, Lidong Miao,Ying Yuan, Xuefeng An; Hubei Province, Caidian District, Wuhan City, Hubei Province People's Hospital, Baojun Hou, Yong Hu; Xingshan County People's Hospital of Hubei Province, Shubing Wu; Jingzhou Central Hospital, Jin Xie, Yaohui Dong; Macheng City People's Hospital, Hongzhuan Cai, Peng Zheng; Changjiang Shipping General Hospital, Xiuqi Li, Han Wang; Zhijiang City People's Hospital, Bing Zhang, Yajie Zhang; Hunan Province, Chenxi County People's Hospital, Xuejin He, Xingguo Tian; Dao County People's Hospital, Shengcheng Zhou, Zhongli Tang; Fenghuang County, Hunan Province People's Hospital, Guangyong Liu, Mingmei Xiao; Hunan Provincial People's Hospital, Xing Wang; Hunan Provincial People's Hospital Mawangdui Hospital, Zhiyi Rong, Wei Luo, Zhineng Zhang; Yongzhou City, Hunan Province Central Hospital, Bin Liu, Guihe Zou, Zheng Guan; Jianghua Yao Autonomous County People's Hospital, Rongjun Wan, Yinyan Xie; Linxiang City People's Hospital, Xiyuan Zhao, Chao Zhang; Shaoyang County People's Hospital, Kaiyou Wu; Xiangtan County People's Hospital, Xuhui Zuo, Xiaoshan Yang; Xinshao County People's Hospital, Jintang Wang; Xupu County People's Hospital, Yangzhou Liang; Yongxing County People's Hospital, Shengyong Deng, Zhixiong Deng; Yuanling County People's Hospital, Rong Cai, Chao Jing; Jilin Province, Beihua University Hospital, Feng Sun, Shiyu Zheng; Dunhua City Hospital, Fanju Meng, Chunbo Zou; Helong City People's Hospital, Yinglin Cui, Xuefei Yu; Hunchun City Hospital, Lijun Yu, Xin Jin; Jilin Province, Jilin Integrative Medicine Hospital, Jianping Shi, Lei Wang, Chengzu Shen; Jilin Provincial People's Hospital, Yuming Du, Yanli Reng, Wei Guo; Yitong County People's Hospital of Jilin Province, Haifeng Wang; Jingyu County People's Hospital, Yuhui Lin, Dehai Jiao; Liaoyuan Second People's Hospital, Aimin Zhang, Yongfen Kang, Lihong Huang; Liaoyuan City Central Hospital, Fenghua Wu; Jiangsu Province, Haimen People's Hospital, Jie Wu, Bin Xu; Nanjing First Hospital, Shaoliang Chen, Hang Zhang; Nantong City Maternal and Child Health Hospital, Song Chen, Jian Chen; Rudong County People's Hospital, Dongmei Liu, Xiaoping Kang, Xiaohong Kang; Jiangxi Province, Chongren County People's Hospital, Chun Yuan, Jiping Wang; Guangchang County People's Hospital, Xiang Fu, Niangfa Wen; Fuzhou, Jiangxi Province Linchuan People's Hospital, Youzhi Zhan; Ji'an City, Jiangxi Province People's Hospital, Xueqiao Wang; Luxi County People's Hospital of Jiangxi Province, Feilong Duan, Xinyu Zhang; Jiangxi Provincial People's Hospital, Qing Huang, Xiaohe Wu; Liaoning Province, Anshan City Double Hill Hospital, Rui Xiao, Xiaoming Gang; Anshan Mayor Hospital, Xiang Jin, Ting Cai; Beipiao Central Hospital, Han Yu, Congwei Huo, Wei Feng; Changtu County First Hospital, Mingbao Sun, Wei Zhang; Zhongshan Hospital Affiliated to Dalian University, Qin Yu, Qianru Bai; Dalian Fifth General Hospital, Haiyan Shao, Jing Zhang; Dalian Central Hospital, Yongchao Zhi, Lili Sun; Dashiqiao Central Hospital, Juan Huang, Qiang Zhang; Liaoyang City Central Hospital, Yingying Li; Yuhong District, Shenyang City People's Hospital, Meijuan Piao, Lili Xin; Inner Mongolia, Alxa League Hospital, Shiguo Hao, Xiaobao Liu; Baotou Fourth Hospital, Baohong Zhang, Conghong Shi; Hellinger County People's Hospital, Yongshuan Wu, Qiuli Wang, Zhiqiang Sun; Keshiketeng Banner Hospital, Lize Wang, Chen Yan, Jian Chen; Hohhot, Hohhot Saihan District Second People's Hospital, Rongjuan Zhang; Inner Mongolia Siziwangqi People's Hospital, Hongtu Zhang, ShuJiang Wang; Tongliao City Horqin District First People's Hospital, Junping Fang, Xinli Yu; Wuhai People's Hospital, Zhaohai Zhou, Lei Shi; Wulateqianqi People's Hospital, Jinlan Xu, Dandan Wang; Wulanchabu City Central Hospital, Dajun Liu, Xinhong Cao; Zhuozi County People's Hospital, Julong Hao, Chunwang Ren; Ningxia, Guyuan Yuanzhou District People's Hospital, Xiaoping Gao, Lining You; Qinghai Province, Qinghai Red Cross Hospital, Yanmei Shen, Xiao Hu; Qinghai Province Fifth People's Hospital, Hong Wu; Zaduo County People's Hospital of Qinghai Province, Cairen Nima, Wangzha Chenglin;

Xining Third People's Hospital, Qing Feng, Jiao Wang; Xinghai County People's Hospital, Guohui Zhou; Shandong Province, Heze City Hospital, Wentang Niu, Sixia Feng; Jining City People's Hospital, Chuanxin Li, Binbao Xiao; Heze City, Shandong Province Chengwu County People's Hospital, Fengqin Liu, Lijuan Wang; Shanxi Province, Huairen County People's Hospital, Ling Tong; Ningwu County People's Hospital, Junhu An; Qinshui County People's Hospital, Hehua Zhang, Yong Gao; Lucheng People's Hospital of Shanxi Province, Yunke Zhou, Xiaoxia Niu; Pianguan County People's Hospital of Shanxi Province, Jinsong Jiao; Xing County, Shanxi Province People's Hospital, Aiping Lv, Yan Zhao; Yuncheng City, Shanxi Province Central Hospital, Bo Wang, Yingjia Li, Zhuoxuan Yang; Zuo Yun County People's Hospital of Shanxi Province, Ru Duan, Xiaolin Li; Taiyuan Xinghualing District Central Hospital, Yueli Qu, Zhimei Yang, Xiaoming Wei; Yanggao County People's Hospital, Zhiru Peng, Yan Han, Hongxia Zhang; Ying County People's Hospital, Wenbing Zhao; Shaanxi Province, Fugu County People's Hospital, Ruijun Hao; Huayin City People's Hospital, Aiping Wang, Feipeng Li; Tongchuan Mining Central Hospital, Guojiong Jia, Huiping Yang; Xi'an First Hospital, Yuqiang Ji, Xia Li; Yangling Demonstration Area Hospital, Xiaoqiang Yang, Honglei Zhang; Shanghai, Shanghai Jiao Tong University School of Medicine Ruijin Hospital, Xiaoxiang Yan; Sichuan Province, Aba Tibetan and Qiang Autonomous Prefecture People's Hospital, Bo Cai, Fangan Li; Guangyuan First People's Hospital, Tianxun Wang, Xiaoying Wang; Muli Tibetan Autonomous County People's Hospital, Hui Peng; Daofu County People's Hospital of Sichuan Province, Jiekang Liu, Lamu Nima; Guang'an Huayang City People's Hospital of Sichuan Province, Zhihong Zhang; Xuanhan County People's Hospital, Xuan Ma, Guochun Jin; Fourth People's Hospital of Zigong City, Yong Yi; Tianjin, Tianjin Jinghai County Hospital, Yuling Zhang, Yan Hua; Tianjin Medical University General Hospital, Yuemin Sun, Bo Bian; Tibet, Gyantse People's Hospital, Ouzhu Danzeng, Ge Sang, Pu Pian ; Xinjiang, Bortala Mongol Autonomous Prefecture People's Hospital, Ping Chen, Edina Cullens; Yunnan Province, Gongshan Dulong Nu Autonomous County People's Hospital, Xiaoping Wu, Yanmin He; Jinning County People's Hospital, Lihua Gu; Lanping Bai Minority Autonomous County People's Hospital, Runxiang He, Jinwen He; Menglian Dai Lahu and Wa Autonomous County People's Hospital, Xiang Li; Qujing Qilin District People's Hospital, Fuyong Li, Yingshuang Yuan, Yuchun Zhang; Yunlong County People's Hospital, Jianxun Yang, Song Ai, Baolong Wang; Zhejiang Province, Dongyang City People's Hospital, Liang Lu, Tingying Xu; Haiyan County People's Hospital, Chunhui Xiao, Zhihua Lu; Huzhou Nanxun People's Hospital, Weili Jin,Fuqin Zhu; Jiaxing Nanhu District Central Hospital East New Campus, Zhihua Sun; Yueqing People's Hospital, Xudong Yu, Qiu Wang; Quzhou City People's Hospital, Xiaoming Tu; Shengsi People's Hospital, Songguo Wang; Taizhou Hospital of Zhejiang Province, Yafei Mi, Weiwei Zhou, Jianjun Jiang; Wencheng County People's Hospital of Zhejiang Province, Junlu Wang, Haisheng Zhu; Yuyao City, Zhejiang Province People's Hospital, Lailin Deng, Lian Chen.

References

1. Zhang Y, Zhang J, Butler J, et al. Contemporary Epidemiology, Management, and Outcomes of Patients Hospitalized for Heart Failure in China: Results From the China Heart Failure (China-HF) Registry. *Journal of cardiac failure.* 2017;23(12):868-875.
